# Characteristics of peripheral Vγ2Vδ2 T cells in interferon-γ release assay negative pulmonary tuberculosis patients

**DOI:** 10.1186/s12879-018-3328-x

**Published:** 2018-09-04

**Authors:** Liping Yan, Hongbo Shen, Heping Xiao

**Affiliations:** Department of Tuberculosis, Shanghai Pulmonary Hospital, Tongji University School of Medicine, 507 Zhengmin Road, Shanghai, 200433 China

**Keywords:** Pulmonary tuberculosis, Vγ2Vδ2 T cells, Interferon-γ release assays, T-SPOT.*TB*

## Abstract

**Background:**

It is not fully explained why some active tuberculosis patients show negative interferon-γ release assays (IGRAs). In this study, we tried to explore associations of IGRAs with the characteristics of peripheral Vγ2Vδ2 T cells and their functions of producing cytokines.

**Methods:**

32 pulmonary tuberculosis patients were enrolled and divided into two groups according to their IGRAs results: 16 with IGRA-negative as test group and 16 with IGRA-positive as control group. Chest X-rays and *T-SPOT.TB* tests were performed and the severity of the lung lesions was scored. The amount of Vγ2Vδ2T cell and their expression levels of the apoptosis-related membrane surface molecule Fas and FasL in peripheral blood were analyzed by flow cytometry, and the function of secreting cytokines (IFN-γ, TNF-α and IL-17A) of Vγ2Vδ2 T cell were determined by intracellular cytokine staining.

**Results:**

The IGRA-negative TB patients had more lesion severity scores and displayed reduced peripheral blood Vγ2Vδ2 T cell counts (*p* = 0.009) as well as higher Fas and FasL expression in peripheral blood Vγ2Vδ2 T cells (*p* = 0.043, 0.026). A high lesion severity score was correlated with a decreased Vδ2+ T cell number and increased Vγ2Vδ2 T cells Fas/FasL expression leve in the peripheral blood (*p* = 0.00, *P* < 0.01). The function of secreting cytokines was slightly impaired in IGRA-negative TB patients (*p* = 0.402). There is no significant differences in expression levels of Fas and FasL in CD4+ T cells (*p* = 0.224, 0.287) or CD8+ T cells (*p* = 0.184, 0.067) between test and control groups.

**Conclusion:**

Compared with IGRA-positive TB patients, the IGRA-negative TB patients had more lesion severity scores, the number of Vγ2Vδ2 T cells decreased and the function of secreting cytokines impaired. In addition, we suggest that increased expression of Fas/FasL triggers Vγ2Vδ2 T cell apoptosis.

**Electronic supplementary material:**

The online version of this article (10.1186/s12879-018-3328-x) contains supplementary material, which is available to authorized users.

## Background

Because of the lack of effective diagnostic methods, particularly in cases of extrapulmonary TB (EPTB), tuberculosis (TB) has the highest mortality rate among all infectious diseases [1, 2]. Interferon-γ release assays (IGRAs) based on the level of IFN-γ secreted by T cells in response to *M. tuberculosis* (MTB) specific antigens including early secreted antigenic target (ESAT-6) and culture filtrate protein (CFP-10) has higher specificity and sensitivity than the conventional tuberculin skin test (TST) [3, 4]. These specific antigens are present in the genome of MTB and absent in the *Bacillus Calmette-Guérin* (BCG) vaccination or most of *non-tuberculous Mycobacteria* (NTM) species [5, 6]. Although studies have observed that IGRA has a high sensitivity varies from 64 to 92% in active TB (ATB) [7, 8] and a number of studies have evaluated factors lowering sensitivity of IGRAs for tuberculosis [9–13], the real cause of ATB with negative IGRAs is far from fully understood.

Some studies using intracellular staining for cytokines suggest that MTB-activated CD4+ and γδ T-cell secreted large amounts of IFN-γ. γδ T cells have been shown to be more potent producers of IFN-γ than CD4+ T cells [14, 15]. γδ T cells, which account for 1–5% of all peripheral blood T cells [16–18] constitute a specific subtype of T cells expressing γδ T cell receptors (TCR) and are referred to as “non- classical” T cells [19]. In particular, Vγ9Vδ2 (also named Vγ2Vδ2) T cells, 60–95% of total circulating γδ T cells, only present in humans and nonhuman primates and remain the sole γδ T-cell subset capable of recognizing phosphor-antigens of MTB [20–22]. The phosphor-antigens of MTB could induce the expansion and expression of functional cytokines of Vγ2Vδ2 T cells [23–26]. Some active pulmonary tuberculosis patients exhibit an decreased ability of Vγ2Vδ2+ T cells to generate IFN-γ in response to phosphor-antigens [27, 28]. Other researches showed that apoptosis is the main reason for the decrease of Vγ2Vδ2 T cells in the peripheral blood of tuberculosis patients [29, 30]. Fas and FasL are apoptosis membrane surface molecules and the Fas/FasL pathway has been shown to be in relation to apoptosis of γδT cells [31]. In our previous study, we have found that anergic pulmonary tuberculosis is accompanied by reduced Vγ2Vδ2 T cell percentage, and elevated Vγ2Vδ2 cell FasL expression [32].

In the present study, we sought to further explore associations of IGRAs with the amount of Vγ2Vδ2 T cells and their functions of producing cytokines, and try to identify factors affecting immunological damage and protection, thereby providing the basis for immunological therapies of tuberculosis.

## Methods

### Patients

The subjects included in this study were hospitalized pulmonary tuberculosis patients in Shanghai Pulmonary Hospital from January 2016 to January 2017. Each patient underwent Chest X-rays and *T-SPOT.TB* tests. There were a total of 32 cases in this study, including 21 men and 11 women, with a body mass index > 18.5 kg/m^2^ The mean age was 43 ± 14 years (range 19–68). The inclusion criteria in this study were the confirmed M. tuberculosis infection using the MGIT BACTEC 960 culture method prior to the treatment. History of tuberculosis, history of taking any immunosuppressive agents including glucocorticoid, serious liver, immune, or kidney diseases, diabetes or tumors, and HIV-positive status were the exclusion criteria. The included pulmonary tuberculosis subjects were divided into two groups based on the results of *T-SPOT.TB* tests and tuberculin skin tests (TST). The test group consisted of 16 patients with negative *T-SPOT.TB* tests and TST results, including 10 men and 6 women, with a mean age of 44 ± 17 years. The control group consisted of 16 pulmonary tuberculosis patients with positive *T-SPOT.TB* tests and TST results. This group included 11 men and 5 women, with a mean age of 43 ± 20 years. The ethics committees of Shanghai Pulmonary Hospital approved the study. Individual participants in the study gave written informed consent before enrollment. The trial was performed in accordance with the Good Clinical Practice guidelines.

### X-ray criteria for the lesion severity scores

Each lung was classified into three zones (upper, middle and lower) and Chest X-rays were divided into six lung fields. The severity of the lung lesions was graded based on the following elements: i) Range of the lung lesions; and ii) number/size of the cavities (outlined in Table 1). The sum of the scores from the six lung fields (every lung field = i + ii) was the final grade of lesion severity. Scores of ≤2.5 were classified as mild, scores between 2.5 and 6 were defined as moderate, and scores ≥6 was classified as severe. Two radiologists and a physician, all of who were highly experienced evaluated all images.

**Table 1:** Criteria for lesion severity

Criteria	Severity score
Disease	
No disease	0
< 50% of area affected	1
≥ 50% of area affected	2
Cavitation	
No cavitation	0
Single cavity	
< 2 cm diameter	0.25
2–4 cm diameter	0.5
> 4 cm diameter	1
Multiple cavities	
Largest < 2 cm diameter	0.5
Largest 2–4 cm diameter	1
Largest > 4 cm diameter	2

### Blood sampling

Prior to the beginning of chemotherapy treatment, 15 mL of anticoagulated peripheral blood sample was collected from each subject.

#### T-SPOT.TB assay

Peripheral blood samples were collected immediately prior to the tests in order to avoid possible interferences. Ficoll-Hypaque gradient centrifugation was used to separate peripheral blood mononuclear cells (PBMCs) at 400×g for 30 min at 20 °C*.* PBMCs were seeded on precoated IFN-γ ELISpot plates and incubated with media containing peptide antigens derived from ESAT-6 (labeled panel A) or CFP-10 (labeled panel B), media without an antigen (as a negative control) in a 5% CO2 atmosphere at 37 °C for 20 h. Phytohemagglutinin was used as a positive control. The number of spot-forming cells was counted. If panels A or B exhibited six or more spots as compared with the negative control, the T-SPOT.TB assay were considered to be positive. The patients who received blood trans- fusions or underwent positron emission tomography-computed tomography scans within 1 week of the test were recommended to undergo a second test two weeks later.

### TST

According to the Mantoux method, 5 tuberculin units of purified protein derivative (PPD) were injected intradermally into the volar aspects of the left forearm. After 72 h, the skin induration was examined. If the induration diameter was≥5 mm, or in the case of blister development, the TST was considered to be positive, while if the diameter was< 5 mm, the TST was considered to be negative.

### Flow cytometry

FITC-TCR Vδ2+ antibodies (BD Bioscience) were used to analyze 100 μl samples of anticoagulated blood from the 32 patients. After 10 μl of Phycoerythrin (PE)- FasL, PE-CY7- Fas, CD3-PB antibodies (BD Bioscience) was added, the whole blood samples were then incubated at room temperature for 30 min in the dark. After 1 ml red blood cell lysis buffer was added, cells were incubated at room temperature in the dark for an additional 15–20 min. After vortexing, the supernatant was discarded and the suspensions were centrifuged at 1400 rpm for 5 min. After using PBS (Protocol Formalin, Kalamazoo, MI) to wash again, the remaining cells were then resuspended in 400 μl PBS. Lymphocyte populations were gated based on the forward and side-scatters on a CyAn ADP flow cytometer (Dako Cytomation, Carpinteria, CA). Gated events were analyzed using Summit Data Acquisition and Analysis Software (Dako Cytomation)(Additional files 1, 2, 3 and 4).

### Intracellular cytokine staining

PBMC were incubated with medium in presence of CD28 (1 μg/ml) and CD49d (1 μg/ml) mAbs in a 200 μl final volume at 37 °C, 5% CO2 for one hour, followed by five-hour incubation in the presence of brefeldin A (GolgiPlug, BD). After a total of six hours incubation, cells were for surface and intracellular staining. In surface staining, cells were incubated with CD3-PBCD4-BV510, CD8-AF700, Vδ2-FITC solutions for 30 min at 4 °C without Ag stimulation in vitro. And then, cells were washed, fixed, permeabilized with FACS perm buffer (PBS supplemented with 0.5% Bovine Serum Albumin-BSA, 0.5% of saponin and 0.1% sodium azide) and incubated in solutions with anti-IFN-γ-BV711, anti-TNF-α-PE-CY7 and anti-IL-17A-PE (BD Biosciences - San Jose, CA, USA) for 30 min at 4 °C. The preparations, then maintained in 200 μL of FACS fix solution until acquisition in a Becton Dickinson FACS calibur instrument (Additional file 5). Isotype IgG was used as negative controls for staining cytokines or surface markers.

### Statistical analyses

The data is presented as mean (x) ± standard deviations (SD). The statistical software SPSS19.0 was applied for analysis. Mean comparisons between groups were performed by using by using Mann-Whitney U test or ANOVA. The distribution analysis was performed by using *Pearson’s X*^*2*^ test. *p* values < 0.05 were considered statistically significant.

## Results

### Demographic profiles of subjects

To explore the relation between IGRAs and Vγ2Vδ2 T cells, we have enrolled two groups of pulmonary tuberculosis patients with IGRAs positive and negative, respectively. The total number of men was 1.9 times that of the women. There were no statistical differences between the two groups of patients regarding age, sex ratio, and body mass index. Therefore, the two groups have demographical comparability (Tables 2 and 3).

**Table 2:** Demographic and clinical characteristics of enrolled subjects in this study

	Test group (*n* = 16)	Control group (*n* = 16)	*P*
Age, years	44.2 ± 17.4	43.9 ± 23.3	*p* = 0.975
Gender (numbers)			
Male (%)	10 (62.5%)	11 (68.8%)	*P* = 0.941
Female (%)	6 (37.5%)	5 (31.2%)	
BMI (kg/m^2^)	21.2 ± 3.4	21.9 ± 5.8	*P* = 0.194
With EPTB (%)	10 (62.5%)	9 (56.25%)	*P* = 0.719

**Table 3:** Study results of the two groups

	Test group (*n* = 16)	Control group (*n* = 16)	*P*
numbers of Vδ2+ T cells	2.40 × 107/L	8.54 × 107/L	*P* = 0.009
Vδ2+ T Fas expression	3.39%	0.91%	*P* = 0.026
Vδ2+ T FasL expression	0.65%	0.55%	*P* = 0.043
CD8 + T Fas expression	13.9%	12.8%	*P* = 0.184
CD8 + T FasL expression	0.65%	0.55%	*P* = 0.067
CD4 + T Fas expression	35.0%	32.8%	*P* = 0.224
CD4 + T FasL expression	0.73%	0.63%	*P* = 0.287
IFN-γ producing Vδ2 + T	0.18%	1.45%	*P* = 0.110
TNF-αproducing Vδ2 + T	0.39%	1.64%	*P* = 0.224
IL-17A producing Vδ2 + T	0.03%	0.15%	*P* = 0.402

### Lesion severity scores between the two groups

Based on the lesion severity scores determined by chest x-rays, we found that 62.5% of the IGRA-negative TB patients had “severe” lesions; 25.0% had “moderate” lesions and 12.5% “mild” lesions. The percentage of the IGRA-positive TB patients who had “severe” lesions was 25.0%. The percentage with a severity score of “moderate” was 25.0% and the percentage of “mild” 50.0%, which was higher than the percentages of patients with either “moderate” or “severe” scores. The comparison of lesion severity scores between the two groups showed statistically significant differences (*p* = 0.045). In summary, the IGRA-negative TB patients had more lesion severity scores.

### Correlation between lesion severity scores and Vδ2+ T cell numbers in the peripheral blood

**Fig. 1:**
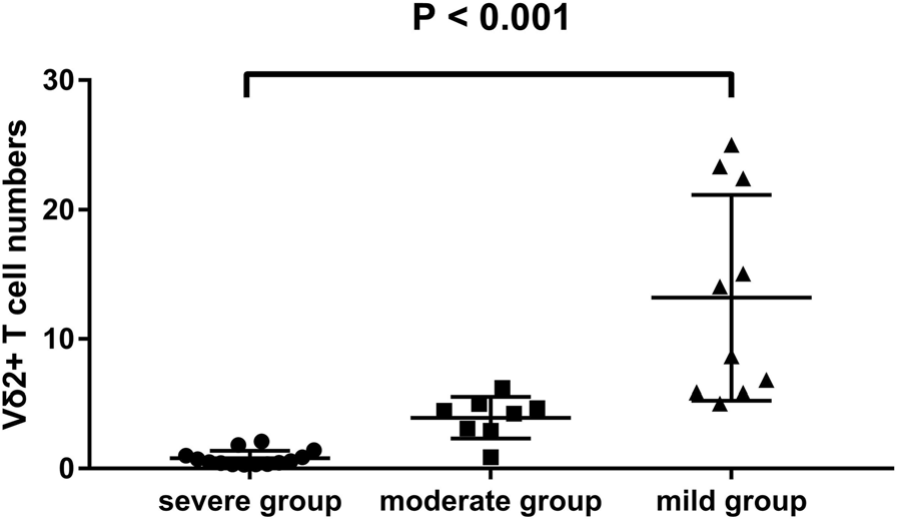
Numbers of Vγ2Vδ2 T cells in peripheral blood of the different lesion groups. In the “mild”, “severe” and “moderate” lesions group, the average Vδ2+ T cell numbers in the peripheral blood was 13.22 (12.08, 13.87) × 107/L, 0.81 (0.65, 1.13) × 107/L and 3.94 (3.09, 5.87) × 107/L

All tuberculosis patients were divided into three subgroups: mild, moderate and severe. Ten patients had “mild” lesions, and in these patients, the average Vδ2+ T cell numbers in the peripheral blood was 13.22 (12.08, 13.87) × 107/L. The numbers of patients who had “severe” lesions was 14 and the corresponding Vδ2+ T cell numbers in the peripheral blood was 0.81 (0.65, 1.13) × 107/L. The average Vδ2+ T cell numbers in the moderate category, which included 8 patients, was 3.94 (3.09, 5.87) × 107/L. The comparison of mean percentage value among the three groups showed statistical differences (*p* = 0.00) (Fig. 1). The more severe the lesions, the lower the Vδ2+ T cells in the peripheral blood count. In a word, a high lesion severity score was associated with a low Vδ2+ T cell count in the peripheral blood.

### Numbers of Vγ2Vδ2 T cells decreased in peripheral blood of IGRA-negative TB patients

**Fig. 2:**
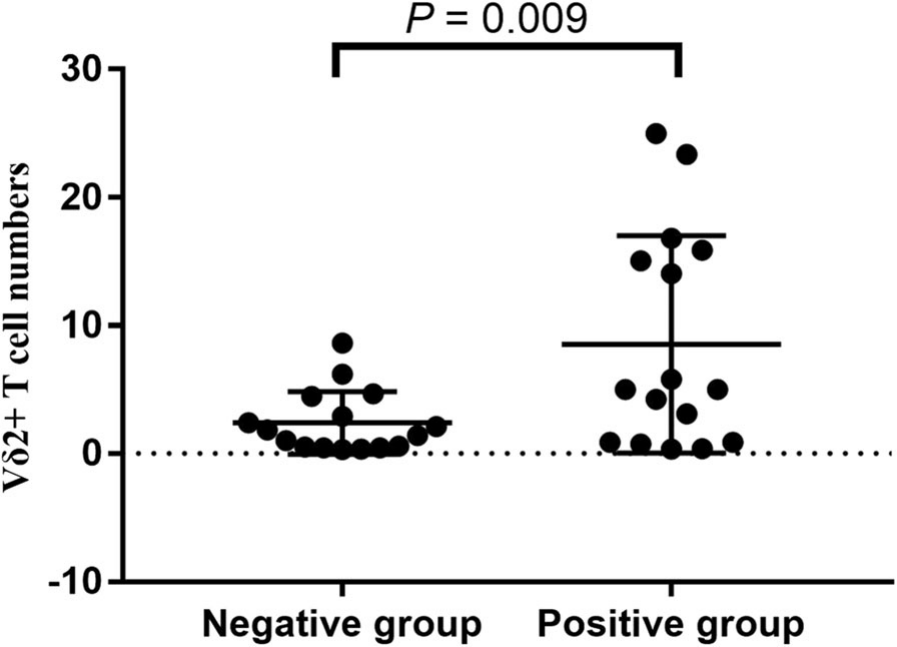
Numbers of Vγ2Vδ2 T cells in peripheral blood of the two groups. The average number of Vγ2Vδ2T in peripheral blood of IGRA- and IGRA+TB patients was 2.40 × 107/L and 8.54 × 107/L

We have compared the number of Vδ2+ T cells among these two groups and found that the average number of Vγ2Vδ2T in peripheral blood of IGRA-negative TB patients and IGRA-positive TB patients was 2.40 (1.28, 3.67) × 107/L and 8.54 (4.78, 12.62) × 107/L, respectively. The peripheral blood Vδ2+ T cell in IGRA-negative TB patients was significantly lower than in IGRA-positive TB patients (*p* = 0.009) (Fig. 2).

### Vγ2Vδ2 T cells highly expressed Fas/FasL in peripheral blood of IGRA-negative TB patients

**Fig. 3:**
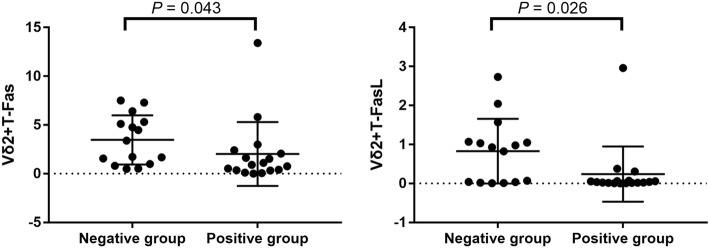
The expression of Fas/FasL in Vδ2+ T cells of the two groups. Vδ2+ T cell Fas expression levels in peripheral blood of IGRA- and IGRA+TB patients were 3.39% and 0.91%. Vδ2+ T cell FasL expression levels in peripheral blood of IGRA- and IGRA+e TB patients were 0.65% and 0.55%

To identify the characteristics Vδ2+ T cells in IGRA-negative patients, we compared the expression of Fas and FasL, which are related with cell apoptosis, in Vδ2+ T cells between two groups. Flow cytometry analyses indicted that Fas expression levels of Vδ2+ T cell in peripheral blood of IGRA-negative TB patients and IGRA-positive TB patients were 3.39% (0.99%, 5.30%) and 0.91% (0.34%, 2.23%), respectively. Vδ2+ T cell FasL expression levels in peripheral blood of IGRA-negative TB patients and IGRA-positive TB patients were 0.65% (0.55%, 2.70%) and 0.55% (0.21%, 1.36%), respectively. The difference of Vδ2+ T cell Fas/ FasL expression levels in peripheral blood between the two groups showed highly statistically significant (*p* = 0.026 and 0.043) (Fig. 3). It implies that the decreased Vδ2+ T cell number in IGRA-negative patients might be related with Fas/FasL-mediated induced cell death.

### Correlation between lesion severity scores and Vγ2Vδ2 T cells Fas/FasL expression levels in peripheral blood

Vδ2+ T cell FasL expression levels in peripheral blood of TB patients with “mild” lesions, “moderate” lesions and “severe” lesions were 0.012% (0.009%, 0.032%), 0.044% (0.019%, 0.083%) and 1.15% (0.84%, 1.32%), respectively. Comparison of mean percentage value among the three groups showed statistically significant differences (*P* < 0.01). Fas expression levels of Vδ2+ T cell in peripheral blood of TB patients with “mild” lesions, “moderate” lesions and “severe” lesions were 0.632% (0.5109%, 0.952%), 1.184% (0.924%, 1.803%) and 5.183% (4.862%, 6.322%), respectively. Comparison of mean percentage value among the three groups showed statistically significant differences (*P* < 0.01). In a word, a high lesion severity score was associated with an increased Vγ2Vδ2 T cells Fas/FasL expression levels in peripheral blood. It implies that disease severity might be correlated with levels of apoptosis of Vγ2Vδ2 T cells in the peripheral blood.

### Comparison of CD8 + T cells Fas/FasL expression levels in peripheral blood of IGRA-negative and IGRA-positive TB patients

**Fig. 4:**
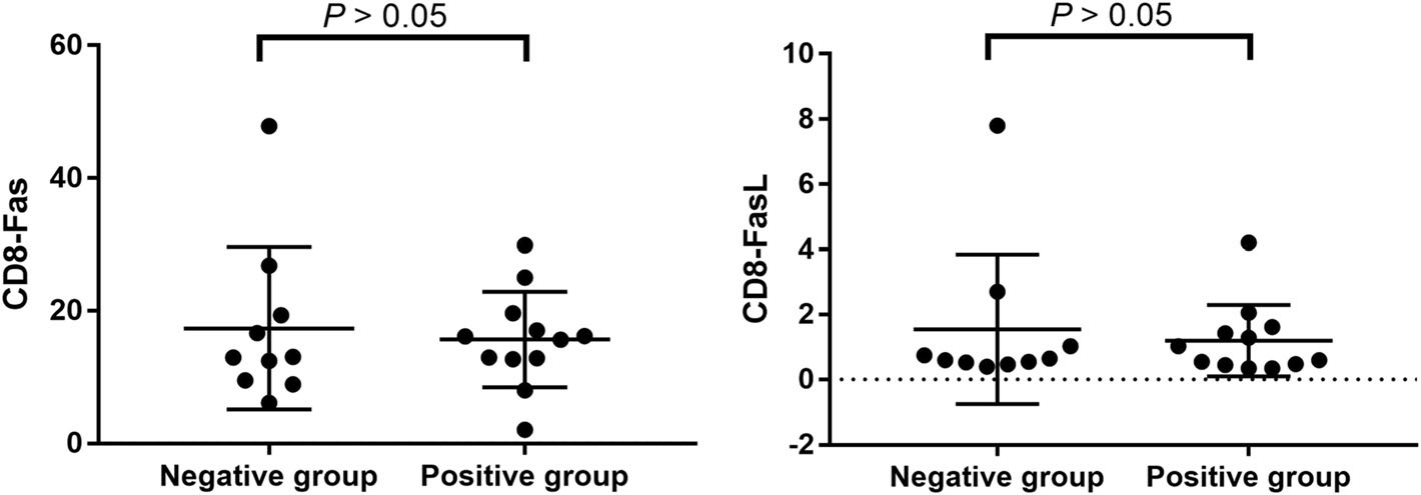
The expression of Fas/FasL in CD8+ T cells of the two groups. CD8+ T cell Fas expression levels in peripheral blood of IGRA- and IGRA+TB patients were 13.90% and 12.80%. CD8+ T cell FasL expression levels in peripheral blood of IGRA- and IGRA+ TB patients were 0.65% and 0.55%

To explore whether the characters of αβ T cells are also different between these two groups, we have tested the expression of Fas/FasL in αβ T cells of TB patients. We found that CD8+ T cell Fas expression levels in peripheral blood of IGRA-negative TB patients and IGRA-positive TB patients were 13.90% (9.89%, 26.80%) and 12.80% (6.15%, 16.6%), respectively. CD8+ T cell FasL expression levels in peripheral blood of IGRA-negative TB patients and IGRA-positive TB patients were 0.65% (0.55%, 2.70%) and 0.55% (0.21%, 1.36%), respectively. However, no statistically significant difference in peripheral blood CD8+ T cell Fas/FasL expression levels was identified between the two groups (*p* = 0.184 and 0.067) (Fig. 4).

### Comparison of CD4 + T cells Fas/FasL expression levels in peripheral blood of IGRA-negative and IGRA-positive TB patients

**Fig. 5:**
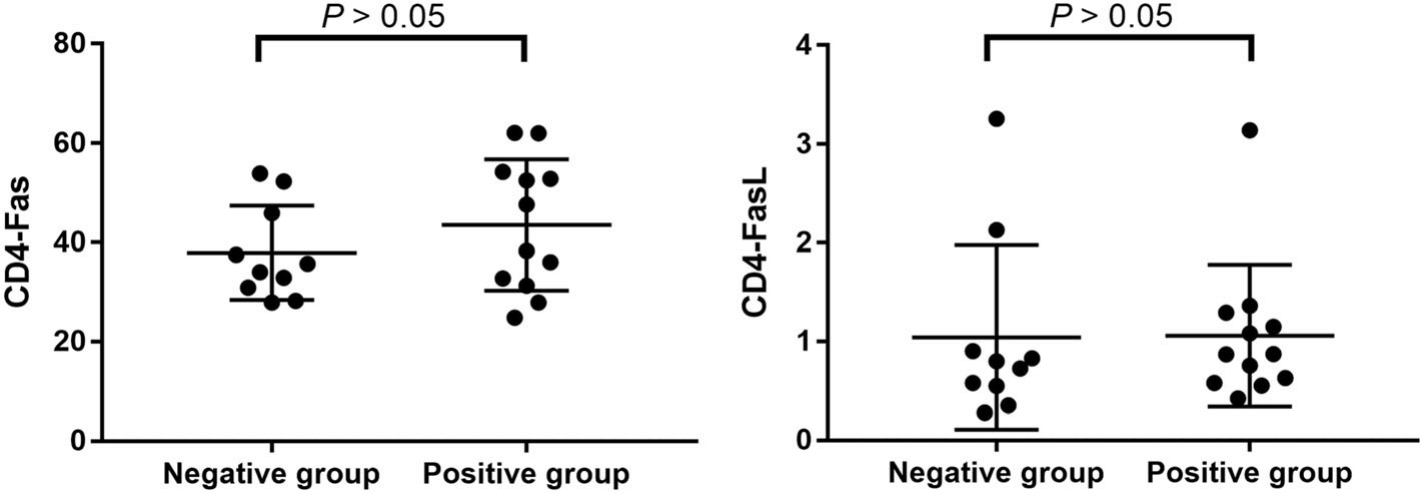
The expression of Fas/FasL in CD4+ T cells of the two groups. CD4+ T cell Fas expression levels in peripheral blood of IGRA- and IGRA+ TB patients were 35.0% and 32.8%. CD4+ T cell FasL expression levels in peripheral blood of IGRA- and IGRA+ TB patients were 0.73% and 0.63%

CD4+ T cell Fas expression levels in peripheral blood of IGRA-negative TB patients and IGRA-positive TB patients were 35.0% (30.9%, 45.9%) and 32.8% (17.8%, 52.7%), respectively. CD4+ T cell FasL expression levels in peripheral blood of IGRA-negative TB patients and IGRA-positive TB patients were 0.73% (0.55%, 2.13%) and 0.63% (0.22%, 1.12%), respectively. However, no statistically significant difference in peripheral blood CD4+ T cell Fas/FasL expression levels was identified between the two groups (*p* = 0.224 and 0.287) (Fig. 5).

### Lower proportion of cytokine producing Vγ2Vδ2 T cells were found in IGRA-negative TB patients

**Fig. 6:**
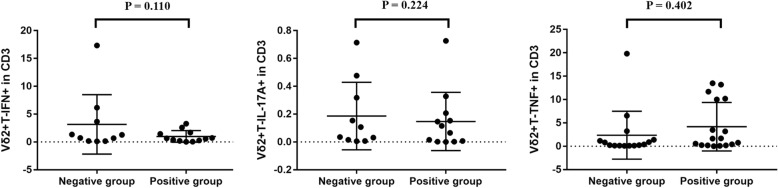
Proportion of cytokine producing Vγ2Vδ2 T cells of the two groups. The proportion of IFN-γ producing Vγ2Vδ2T cells within CD3+ T-cells was 0.18% in peripheral blood of IGRA- TB patients, which was highly lower than 1.45% in IGRA+ TB patients. The proportion of TNF-α producing Vγ2Vδ2T cells within CD3+ T-cells was 0.39% in peripheral blood of IGRA- TB patients, while it was 1.64% in IGRA+ TB patients. The proportion of IL-17A producing Vγ2Vδ2T cells within CD3+ T-cells was 0.03% and 0.15% in peripheral blood of IGRA- and IGRA+ TB patients

To test whether there is difference in ability of Vδ2+ T cells producing functional cytokines in IGRA-negative patients, we compared the expression of IFN-γ, TNF-α and IL-17 in Vδ2+ T cells between two groups. The proportion of IFN-γ producing Vγ2Vδ2T cells within CD3+ T-cells was 0.18% (0.16%, 1.34%) in peripheral blood of IGRA-negative TB patients, which was highly lower than 1.45% (0.33%, 10.2%) in IGRA-positive TB patients. However, no statistically significant difference was observed (*p* = 0.110). The proportion of IL-17A producing Vγ2Vδ2T cells within CD3+ T-cells was 0.39% (0.15%, 1.39%) in peripheral blood of IGRA-negative TB patients, while it was 1.64% (0.22%, 10.11%) in IGRA-positive TB patients. The difference between the two groups showed no statistically significant (*p* = 0.224). The proportion of TNF-α producing Vγ2Vδ2T cells within CD3+ T-cells was 0.03% (0.01%, 0.15%) and 0.15% (0.01%, 0.32%) in peripheral blood of IGRA-negative TB patients and IGRA-positive TB patients, respectively. There was no statistically significant difference (*p* = 0.402) (Fig. 6).

## Discussion

Vγ2Vδ2T cells were reported to play an important role in host immunity resistant to M. tuberculosis [33]. It was showed that Vγ2Vδ2T cells kill intracellular bacilli through killing of macrophages harboring live MTB; moreover, these cells can not only reduces the activity of extracellular MTB, but also can reduces the activity of intracellular MTB. These findings have suggested that Vγ2Vδ2T cells directly play a role in host protective reaction against MTB infection [34]. Previous study showed that Vγ9Vδ2 T cells from children with active TB have a decreased IFN-γ production [35]. Another research showed that the quantity of Vγ9Vδ2 T cells in the peripheral blood of TST-negative tuberculosis patients is dramatic declined [29]. In our previous study, we found that the Vγ9Vδ2 T cells percentage in the peripheral blood of tuberculosis patients with TST negative was significantly lower than in patients with TST positive [32]. Will the amount of Vγ9Vδ2 T cells and cytokine secretion ability decrease in IGRA-negative TB patients? To address this possibility, we conducted the present study. In the present study, we found that the amount of Vγ9Vδ2 T cells in the peripheral blood of IGRA-negative TB patients was significantly lower than in IGRA -positive TB patients. In addition, the percentage of Fas/FasL-expressing Vγ9Vδ2 T cells in the peripheral blood of IGRA-negative TB patients was significantly higher than in IGRA -positive TB patients, suggesting that the decrease in the number of Vγ9Vδ2 T cell may be related to the increase in Fas/FasL-mediated induced cell apoptosis. Li et al. also found that Fas/FasL upregulation was induced and subsequently Vγ9Vδ2 T cell apoptosis increased via co-culturing of M. tuberculosis antigens and γδ T cells in vitro. It is worth mentioning that we also analyzed CD8 + T and CD4 + T cells Fas/FasL expression levels in peripheral blood of IGRA-negative and IGRA-positive TB patients in this study. However, there were no significant differences between IGRA-negative and IGRA-positive TB patients.

It is known that the activation of Vγ9Vδ2 T cells induces the secretion of a variety of cytokines, thereby regulating immune responses. Vγ9Vδ2 T cells can increase host immunity against infection by secreting IFN-γ. Besides IFN-γ, TNF-α is also a key molecule in host immunity to tuberculosis [36, 37]. TNF-α strengthens immune cell migration and orientation in the presence of M. tuberculosis and effects the presentation of adhesion molecules [38]. IL-17A has been found to be a significant cytokine in the immune reaction against mycobacterial infection [39]. Investigations have showed that the IL-17A-producing γδ T cells were main source of IL-17A [40]. Our results revealed that the proportion of cytokine (including IFN-γ, TNF-α and IL-17A) producing Vγ2Vδ2T cells within CD3+ T-cells in peripheral blood of IGRA-negative TB patients was lower than in IGRA-positive TB patients.

Study by Szereday et al. reported that tuberculosis patients with negative TST results often have severe atypical clinical manifestations [41]. In previous study, we found that very few Vδ2+ T cells not only in the peripheral blood but also in BALF of PPD-negative tuberculosis patients [32]. In present study, we observed that a high lesion severity score was correlated with a decreased Vδ2+ T cell number and increased Vγ2Vδ2 T cells Fas/FasL expression leve in the peripheral blood, a phenomenon that was consistent with a previous study by Pinheiro et al., who found that the reduction of peripheral γδ T cell is closely associated with higher lesion severity in tuberculosis patients [42].

## Conclusions

In summary, our research suggests that compared with IGRA-positive TB patients, the IGRA-negative TB patients had more lesion severity scores, the number of Vγ9Vδ2 T cells decreased and the function of secreting cytokines slightly impaired. In addition, we suggest that high expression of Fas/FasL triggers Vγ9Vδ2 T cell apoptosis.

## Additional files

Additional file 1: **Figure S1.** Numbers of Vγ2Vδ2 T cells in peripheral blood of the two groups. (JPG 57 kb)

Additional file 2: **Figure S2.** The expression of Fas/FasL in Vδ2+ T cells of the two groups. (JPG 38 kb)

Additional file 3: **Figure S3.** The expression of Fas/FasL in CD8+ T cells of the two groups. (JPG 45 kb)

Additional file 4: **Figure S4.** The expression of Fas/FasL in CD4+ T cells of the two groups. (JPG 45 kb)

Additional file 5: **Figure S5.** Proportion of cytokine producing Vγ2Vδ2 T cells of the two groups. (JPG 46 kb)

## Abbreviations

ATB: active pulmonary tuberculosis
BCG: Bacillus Calmette-Guérin
CFP-10: culture filtrate protein
CT: computed tomography
EPTB: extrapulmonary
ESAT-6: early secreted antigenic target
IGRAs: interferon-γ release assays
MTB: Mycobacterium tuberculosis
NTM: non- tuberculos mycobacteria
PBMCs: Peripheral blood mononuclear cells
SD: standard deviation
TB: Tuberculosis
TCR: T cell receptors
TST: tuberculin skin test
