# Supplementary figures and images for: Characteristics of peripheral Vγ2Vδ2 T cells in interferon-γ release assay negative pulmonary tuberculosis patients
Source: BMC Infect Dis. 2018 Sep 4;18:453. doi: 10.1186/s12879-018-3328-x (PMC6123966; doi:10.1186/s12879-018-3328-x)

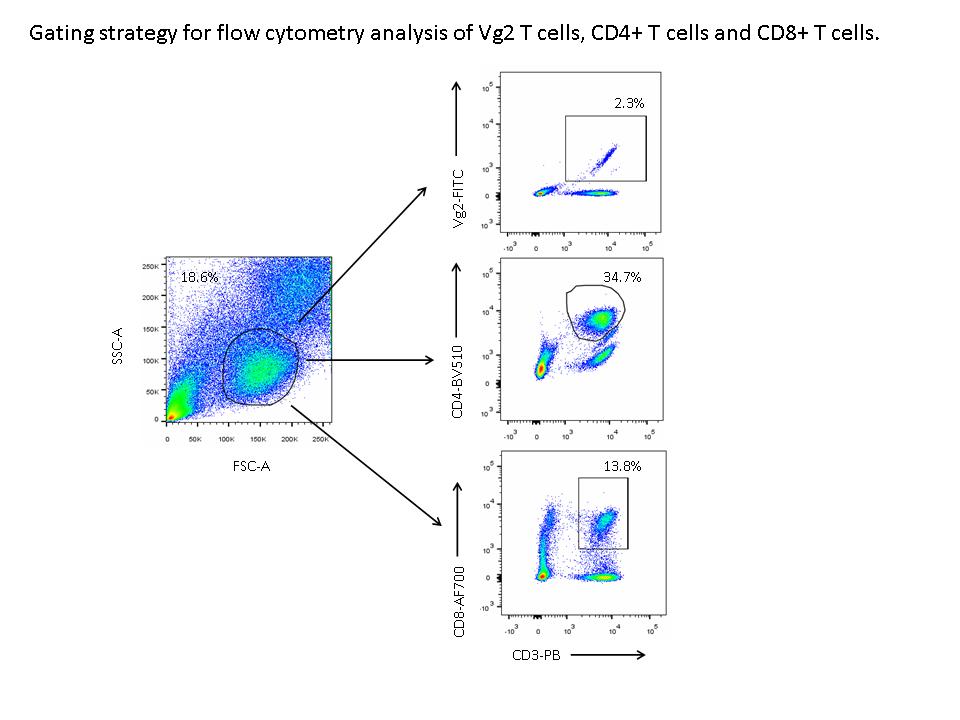

Supplement: Supplementary file 1 — Figure S1. Numbers of Vγ2Vδ2 T cells in peripheral blood of the two groups. (JPG 57 kb) [file 12879_2018_3328_MOESM1_ESM.jpg]

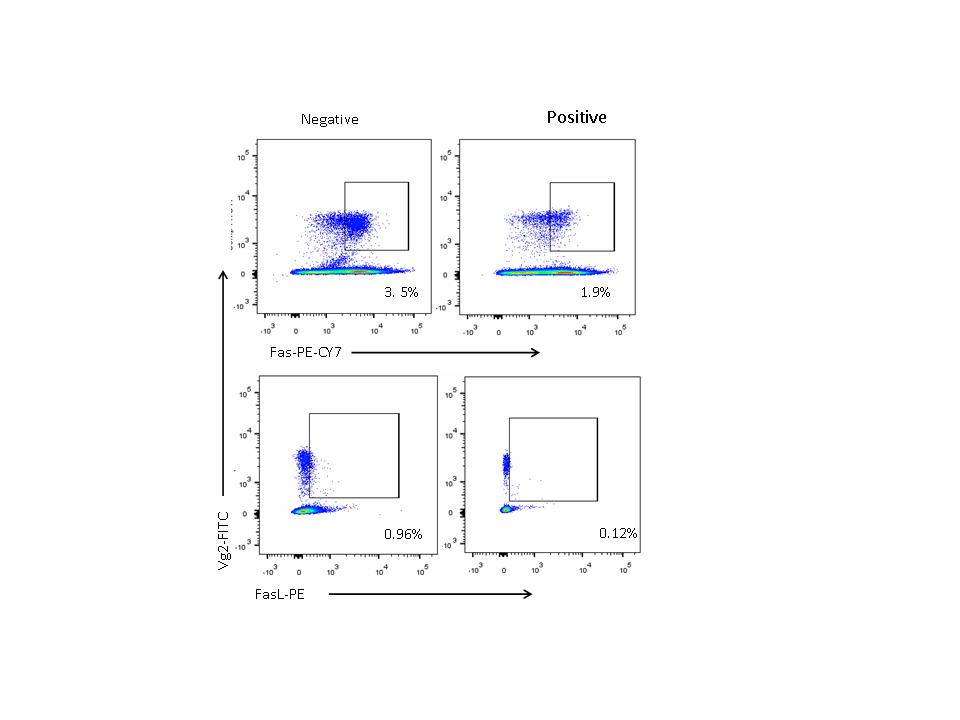

Supplement: Supplementary file 2 — Figure S2. The expression of Fas/FasL in Vδ2+ T cells of the two groups. (JPG 38 kb) [file 12879_2018_3328_MOESM2_ESM.jpg]

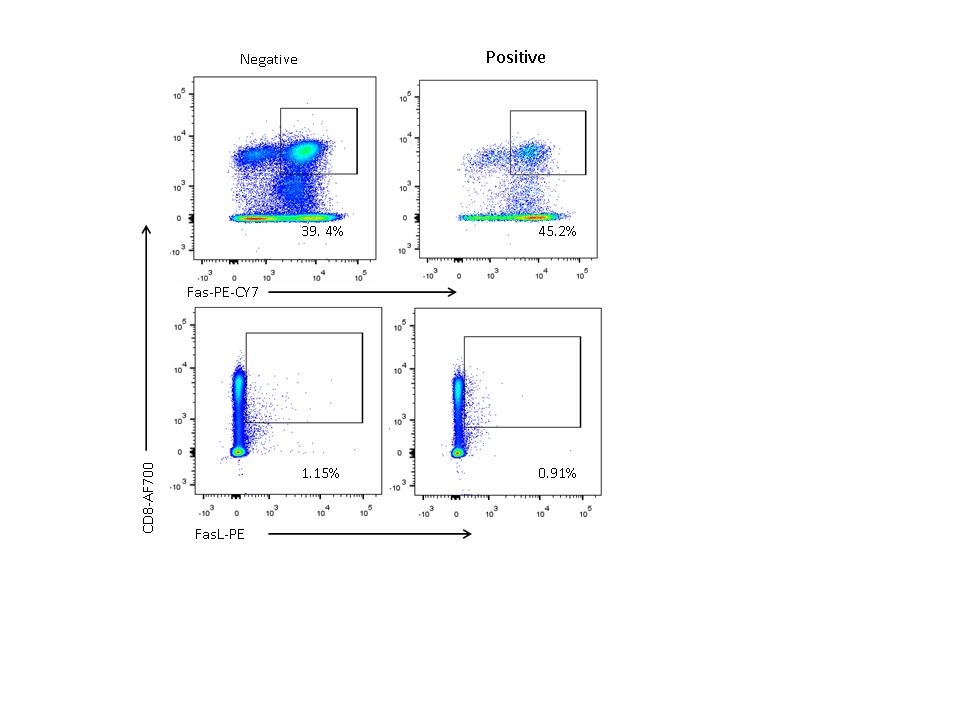

Supplement: Supplementary file 3 — Figure S3. The expression of Fas/FasL in CD8+ T cells of the two groups. (JPG 45 kb) [file 12879_2018_3328_MOESM3_ESM.jpg]

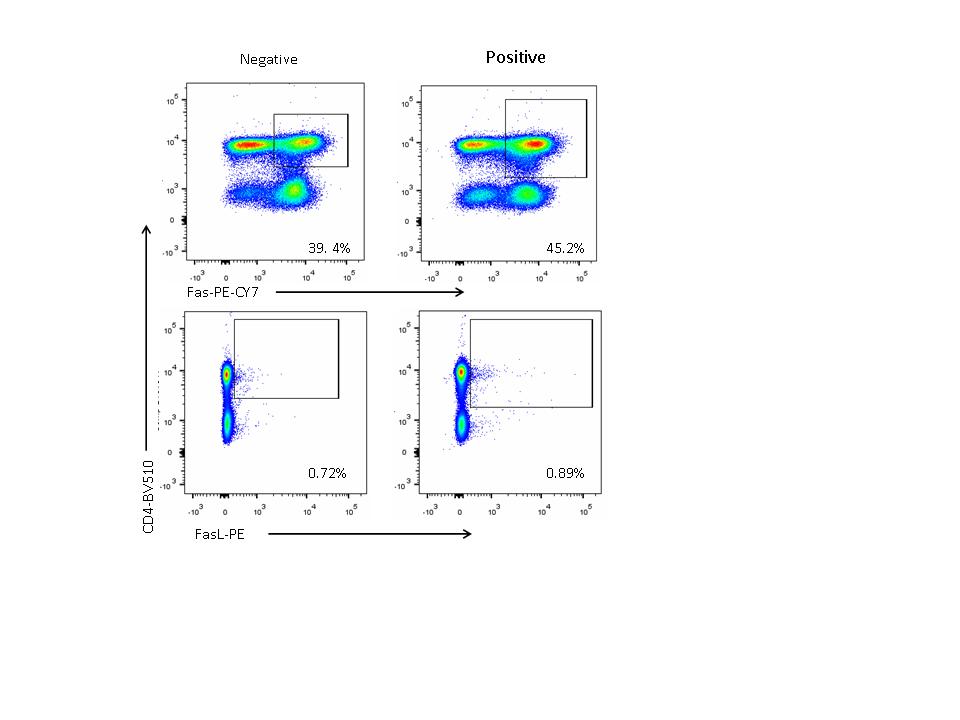

Supplement: Supplementary file 4 — Figure S4. The expression of Fas/FasL in CD4+ T cells of the two groups. (JPG 45 kb) [file 12879_2018_3328_MOESM4_ESM.jpg]

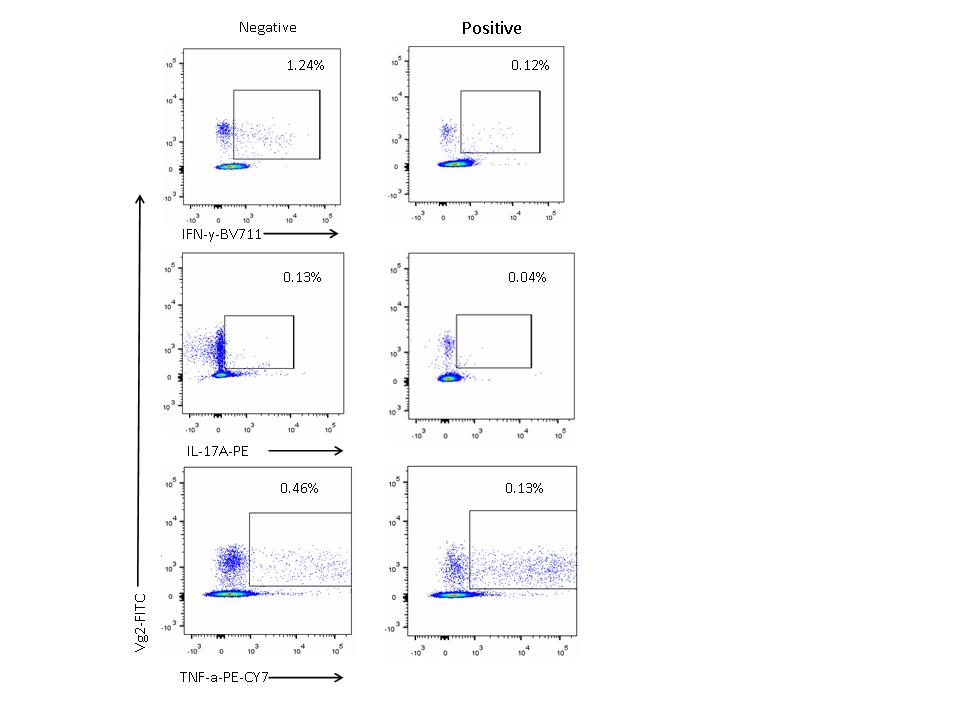

Supplement: Supplementary file 5 — Figure S5. Proportion of cytokine producing Vγ2Vδ2 T cells of the two groups. (JPG 46 kb) [file 12879_2018_3328_MOESM5_ESM.jpg]
